# Extra-Nodal, Nasal, Natural Killer T-Cell Lymphoma Treated With a Checkpoint Inhibitor: A Case Report of a Sustained Complete Response

**DOI:** 10.7759/cureus.14654

**Published:** 2021-04-23

**Authors:** Radwan Diab, Syed Kamran, Bridget Adcock, Khalil Choucair, Quoc V Truong

**Affiliations:** 1 Internal Medicine, University of Kansas School of Medicine, Wichita, USA; 2 Hematology/Oncology, Cancer Center of Kansas, Wichita, USA

**Keywords:** enktl, immune therapy, pd-1 inhibitor, immune checkpoint inhibitor, abscopal effect

## Abstract

Extra-nodal natural killer T-cell lymphoma (ENKTL) is a rare and aggressive hematologic malignancy found in the nasal cavity and adjacent locations in 80% of cases, accounting for approximately 10% of non-Hodgkin lymphoma (NHL) and 0.4% of all cancers. Prognosis is typically poor and depends on stage, location, age, and tumor markers for targeted therapy, which is reserved for relapsed/refractory ENKTL. Of those, advanced clinical stage, higher Prognostic Index (PI), nodal involvement, Ki-67 expression, large cells, local tumor invasiveness, and circulating Epstein-Barr virus (EBV)-DNA levels are predictive of worse survival. Here, we present a rare case of a patient in remission 30 months after diagnosis of ENKTL following a sustained complete response to pembrolizumab, an immune checkpoint inhibitor targeting the programmed death-1 (PD-1) receptor.

## Introduction

Extra-nodal natural killer T-cell lymphoma (ENKTL) is a rare and aggressive hematologic malignancy found in the nasal cavity and adjacent locations in 80% of cases [1]. Most commonly found in East Asia and Latin America, it accounts for approximately 10% of non-Hodgkin lymphoma (NHL) and 0.4% of all cancers. In the United States, Canada, and Europe, prevalence is as low as 1%-5% of all NHL, although there is an increasing incidence in these locations estimated at +0.6% to 1.2% in age-standardized annual percent change between 1993 and 2008 [2]. Other risk factors include male sex, mean age of 50 years or older, and Epstein-Barr Virus (EBV) infection [3].

ENKTL initially presents as a progressive ulceration with necrotic granuloma noted in the nasal cavity, palate, and nasopharynx. Symptoms are often associated with tumor invasion of local tissues such as facial skin, paranasal sinus, and orbits with patients most commonly presenting with nasal obstruction, bloody diarrhea, facial swelling, sore throat, and hoarseness [4]. Other systemic symptoms are also reported at presentation including prolonged fever and weight loss.

Diagnosis of ENKTL is usually confirmed via biopsy [5] and positive immunostaining for CD2, CD56, cytoplasmic CD3 epsilon, and cytotoxic molecules such as granzyme B, TIA1, and EBV-encoded RNA positivity [6]. Histology shows lymphoid infiltrates often causing fibrinoid necrosis [5]. Prognosis depends on stage, location, age, and tumor markers for targeted therapy, which is reserved for relapsed/refractory ENKTL. While higher expression of PD-L1 favors prognosis, strong expression of CD38, alteration in DDX3X gene and STAT3 mutations have been associated with poor outcomes. When comparing localized disease and ENKTL, nasal type tends to have better outcomes than advanced disease and non-nasal type ENKTL. Other adverse prognostic factors include age > 60 years, male sex, distant lymph node involvement, and stage III or stage IV disease. Here we present a rare case of a patient in remission 30 months after diagnosis of ENKTL following a sustained complete response to PD-1 immune checkpoint inhibitor.

## Case presentation

An 82-year-old Asian male presented to his oncologist with the chief complaint of bilateral, painful neck swelling. Onset of symptoms was subacute over a month prior to this presentation. Symptoms were constant and progressively worsening, mainly involving pain and discomfort in the neck area, as well as weight loss. He denied any associated fever or night sweats, dysphagia, hemoptysis, or hematemesis. The remainder review of system was negative, and the patient was only endorsing a feeling of facial fullness. His past medical history was significant for stage IIIB colon cancer and prostate cancer. He had undergone a hemicolectomy followed by FOLFOX (folinic acid + fluorouracil + oxaliplatin) chemotherapy three years earlier achieving complete remission. The patient had also completed radiation therapy for prostate cancer one year earlier and was on leuprolide androgen deprivation therapy (ADT). Social history was only significant for a history of smoking (15 pack-years; quit > 10 years earlier). Family history was non-contributory. Physical examination was significant for bilateral tender cervical swelling, and besides that, vital signs, the remainder of the examination, and laboratory testing were primarily benign. He had been in his usual state of good health and, besides ADT, was only taking celecoxib as a prophylaxis for colon cancer recurrence.

**Figure 1:**
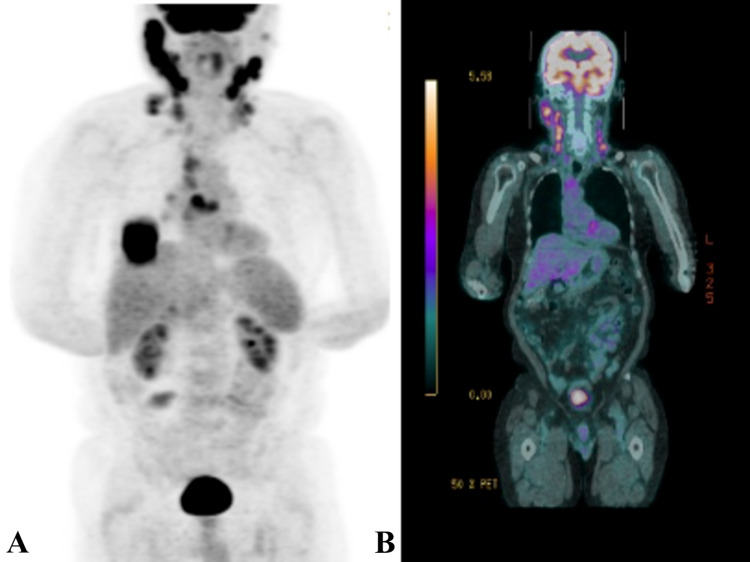
Imaging at diagnosis revealed multiple hot spots reflecting active disease (A) Positron emission tomography (PET) findings at time of ENKTL diagnosis presented as a three-dimension (3D) reconstruction revealing multiple hot spots reflecting active disease. (B) PET-computerized tomography (PET-CT) findings at time of ENKTL diagnosis revealing slice-by-slice sites of active disease. ENKTL, Extra-nodal natural killer T-cell lymphoma.

**Figure 2:**
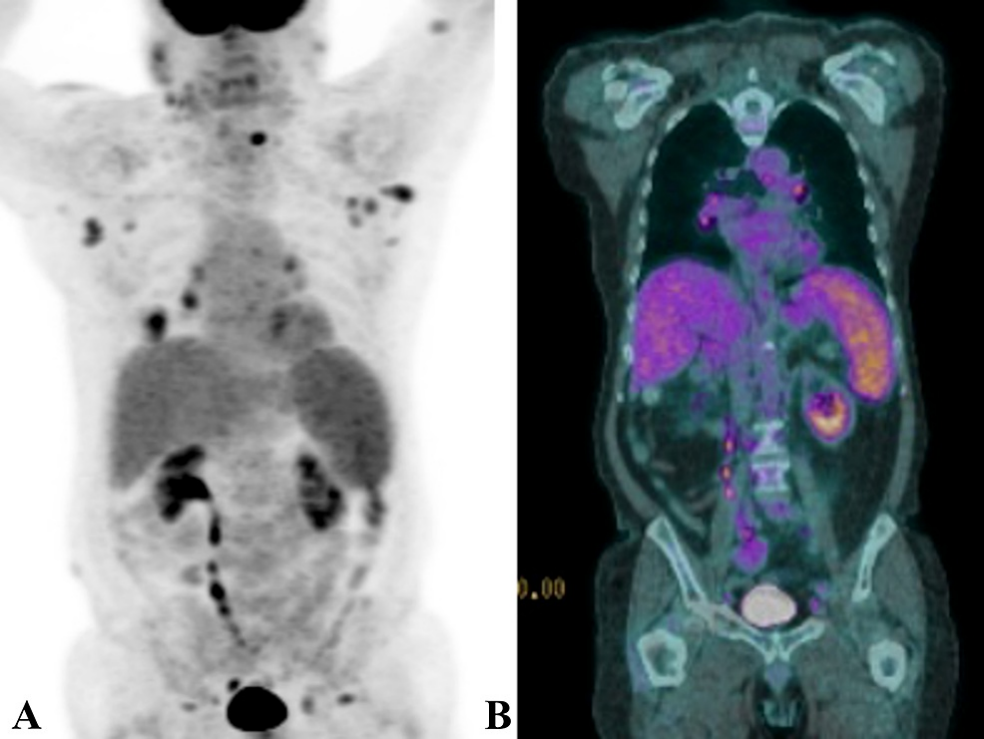
ENKTL relapse following standard of care chemotherapy Radiographic findings at the time of ENKTL relapse following initial treatment with GDP chemotherapy are seen on a (A) positron emission tomography (PET) scan with a three-dimension (3D) reconstruction revealing multiple hot spots reflecting active disease and on a (B) PET-computerized tomography (PET-CT) revealing slice-by-slice sites of active disease. ENKTL, Extra-nodal natural killer T-cell lymphoma.

**Figure 3:**
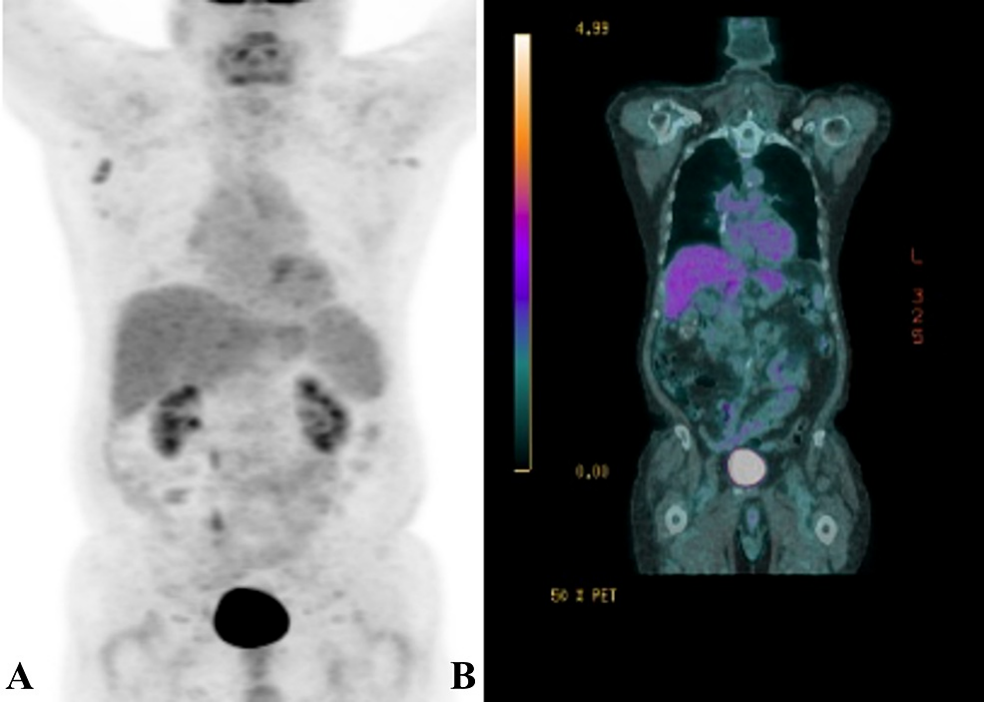
Repeat imaging following pembrolizumab therapy revealed complete radiological response Repeat imaging following two cycles of pembrolizumab ICI therapy using (A) positron emission tomography (PET) scan and (B) PET-computerized tomography (PET-CT) revealing complete radiologic response to therapy. ICI, Immune checkpoint inhibitor.

An initial ultrasound-guided needle core biopsy of the right cervical lymph node revealed partially necrotic lymphoid tissue, CD3+ T-lymphocytes as well as scattered CD20+ B-lymphocytes. Other immunostains to rule out metastatic disease from colon or prostate such as CK AE1/AE3 (carcinoma cell markers), prostate-specific antigen (PSA), and prostate-specific acid phosphatase (PSAP) (for possible prostate metastasis) were negative. Thyroid transcription factor 1 (TTF-1) and thyroglobulin markers of thyroid carcinoma were negative as well. ENKTL-specific immunostains were not ordered on the initial biopsy, given low suspicion of the disease. The initial results were thus inconclusive for either a metastatic carcinoma or lymphoma diagnosis. The patient was referred for an otolaryngology evaluation, and a nasal endoscopy-guided biopsy of the right turbinate established the diagnosis of ENKTL. CT scan with contrast of the neck soft tissue and face as well as positron emission tomography (PET)/CT scans of the chest, abdomen, and pelvis were performed for initial staging. Imaging results revealed involvement of the nasal turbinate, right paranasal sinuses, bilateral cervical lymph nodes, as well as hilar and pulmonary involvement. These findings confirmed the diagnosis of disseminated nasal NK/T-cell lymphoma (Figure 1). His disease was deemed aggressive (stage IV of the tumor node metastasis [TNM] staging system), and chemotherapy with gemcitabine/dexamethasone/cisplatin (GDP) was initiated resulting in near complete radiologic response on four-month PET scans. Repeat PET scans at eight months revealed disease relapse (Figure 2), and a sample from the patient’s original biopsy was sent for next-generation sequencing (NGS) assay analysis to explore possible molecular targets and guide next-line therapy. NGS results revealed mutations in TET2 gene and a positive expression of programmed death ligand-1 (PD-L1) at 15%. Treatment with pembrolizumab (Keytruda®) was thus initiated and well-tolerated with no immune-mediated side effects. PET scan after second cycle of immune therapy revealed complete resolution of disease (Figure 3). At 21 months after immune therapy initiation and 30 months after initial diagnosis, the patient remains in complete remission with no measurable disease.

 

 

## Discussion

We have reported a rare case of aggressive disseminated nasal ENKTL, which initially failed treatment with first-line chemotherapy regimen (GDP) and subsequently experienced a complete response to molecular target PD-1 inhibitor, pembrolizumab, with minimal adverse effects. Although first-line treatment for stage I/II ENKTL is radiotherapy, it is associated with an increased incidence of relapse even for early-stage patients [7]. Some studies show as high as 18.8% relapse with radiotherapy alone [8]. Thus, in stage I/II patients in whom non-anthracycline chemotherapy is feasible, chemotherapy should be coupled with radiotherapy [7]. Concurrent chemoradiotherapy (CCRT) and sequential chemotherapy involving non-anthracycline regimens (L-asparaginase) demonstrated similar results for objective response rate (ORR), complete response (CR) for treatment, overall survival (OS), and progression-free survival (PFS), and either is used as first-line treatment for stage I/II ENKTL [7-9]. In contrast, treatments for advanced stage III/IV and relapsed/refractory ENKTL are not as effective as those for early-stage ENKTL treatments [7]. Hence, there is an urgent need for new combination regimens with increased efficacy and minimal toxicity for stage III/IV ENKTL. Currently, L-asparaginase-containing regimens are considered the standard for treatment for stage III/IV ENKTL [7]. However, overall prognosis and survival remain relatively poor, with five-year OS rates ranging between 45% and 57% [10,11] and a median OS time of eight months as reported by the International Peripheral T-Cell and Natural Kill T-Cell Lymphoma Study [12]. In contrast, our patient has maintained an ongoing 30-month period of CR to immune checkpoint inhibition.

While previous focus had mainly been on chemoradiotherapy, today’s paradigm in the treatment of ENKTL has shifted to targeted immunotherapy. However, the reported survival rates using immunotherapy in ENKTL are variable with most results, either preliminary from clinical trials or anecdotal from case reports. With the exception of brentuximab vendotin (BV), most immunotherapy agents being investigated as targeted therapies for ENKTL are either under phase 1 and/or phase 2 trials [13,14]. In fact, BV achieved CR in two cases of ENKTL with CD30 expression [15]. On the other hand, according to preliminary results of a phase 2 trial (NCT02927925) announced at the 2018 American Society of Hematology meeting, daratumumab promoted 35.7% ORR in refractory/relapsed ENKTL that failed at least one line of chemotherapy [13]. Such encouraging results could be explained by the potentiating effect of immunotherapy with chemotherapy. For example, a six-week course of daratumumab achieved sustained CR for 21 weeks in one case of ENKTL that had relapsed after a six-week course of chemoradiotherapy and allogenic stem cell transplantation [16]. Similarly, a patient with ENKTL had been reported to achieve CR following treatment with bortezomib, fludarabine, and autologous hematopoietic stem cell transplantation [17]. These observations, along with the case we present here, raise the possibility of a potential synergistic effect between chemoradiation and subsequent immune therapy.

Similar to our case report, two other studies (age range: 31-68 years) have reported PD-1 inhibitors’ efficacy against refractory/relapsed ENKTL. For example, treatment with seven cycles of pembrolizumab achieved 100% ORR (including complete response, partial response, and stable disease) and 71.4% CR among seven patients with refractory/relapsed ENKTL [18]. However, unlike our case report, median follow-up was only six months. Additionally, nivolumab (another PD-1 inhibitor) has been reported to induce a complete clinical response for three patients with refractory/relapsed ENKTL [19]. Such encouraging results in patients with disease relapse, who had previously received chemoradiation therapy, could be explained by immunotherapy’s potentiation to the abscopal effect. Although its mechanism is not definitively defined, the abscopal effect is thought to be due to an adaptive immune response targeting neoantigens released from radiotherapy-induced tumor cell lysis [20]. Interestingly, the abscopal effect has been reported when radiation therapy was used as a monotherapy in highly immunogenic tumors, with effects persisting up to 30 months after treatment completion [20]. Hence, the presence of robust adaptive immune system is essential to promote tumor immunogenicity. In this case report, it is reasonable to hypothesize that the patient had comorbid ENKTL lymphoma and benefited from radiotherapy targeting his prostate cancer. Although not intended, it is conceivable that the multiple modalities of treatment this patient had undergone including radiotherapy for prostate cancer, systemic therapies for colorectal cancer, and first-line chemotherapy for ENKTL may have increased the ENKTL’s tumor immunogenicity, allowing a more robust cellular response with subsequent immunotherapy with pembrolizumab [20]. Despite having remission after targeted chemotherapy, this multifaceted approach, in addition to the patient's PD-L1 status, may have hypersensitized patient’s tumor immunogenicity paving the way for pembrolizumab to promote sustained complete remission for 21 months.

## Conclusions

Targeting immune checkpoints has recently showed increasing efficacy in ENKTL patients who have limited treatment options but have positive PD-L1 tumor expression, similar to our patient (15% PD-L1 positivity). Unlike the limited survival benefits reported in the literature with the use of systemic chemoradiation approach, use of checkpoint inhibitors seems to confer added survival benefit. In our case, the patient even exceeded the reported survival rates with use of PD-1/PD-L1 inhibitors and has been in remission for 30 months. It is conceivable that prior systemic treatment may have increased sensitivity to immune therapy resulting in prolonged survival. However, further larger trials are needed to clearly establish the role of immune checkpoint-targeted therapies in advanced ENKTL, either alone or in a therapeutic sequence.
